# Use of HbA1c in the Identification of Patients with Hyperglycaemia Caused by a Glucokinase Mutation: Observational Case Control Studies

**DOI:** 10.1371/journal.pone.0065326

**Published:** 2013-06-14

**Authors:** Anna M. Steele, Kirsty J. Wensley, Sian Ellard, Rinki Murphy, Maggie Shepherd, Kevin Colclough, Andrew T. Hattersley, Beverley M. Shields

**Affiliations:** 1 NIHR Exeter Clinical Research Facility, University of Exeter, Exeter, Devon, United Kingdom; 2 Research and Development, Royal Devon and Exeter NHS Foundation Trust, Exeter, Devon, United Kingdom; 3 Department of Molecular Genetics, Royal Devon & Exeter NHS Foundation Trust, Exeter, Devon, United Kingdom; 4 Department of Medicine, Faculty of Medical and Health Sciences, University of Auckland, Auckland, New Zealand; Emory Univ. School of Medicine, United States of America

## Abstract

**Aims:**

HaemoglobinA1c (HbA1c) is recommended for diabetes diagnosis but fasting plasma glucose (FPG) has been useful for identifying patients with *glucokinase* (*GCK*) mutations which cause lifelong persistent fasting hyperglycaemia. We aimed to derive age-related HbA1c reference ranges for these patients to determine how well HbA1c can discriminate patients with a *GCK* mutation from unaffected family members and young-onset type 1 (T1D) and type 2 diabetes (T2D) and to investigate the proportion of *GCK* mutation carriers diagnosed with diabetes using HbA1c and/or FPG diagnostic criteria.

**Methods:**

Individuals with inactivating *GCK* mutations (n = 129), familial controls (n = 100), T1D (n = 278) and T2D (n = 319) aged ≥18years were recruited. Receiver Operating Characteristic (ROC) analysis determined effectiveness of HbA1c and FPG to discriminate between groups.

**Results:**

HbA1c reference ranges in subjects with *GCK* mutations were: 38–56 mmol/mol (5.6–7.3%) if aged ≤40years; 41–60 mmol/mol (5.9–7.6%) if >40years. All patients (123/123) with a *GCK* mutation were above the lower limit of the HbA1c age-appropriate reference ranges. 69% (31/99) of controls were below these lower limits. HbA1c was also effective in discriminating those with a *GCK* mutation from those with T1D/T2D. Using the upper limit of the age-appropriate reference ranges to discriminate those with a mutation from those with T1D/T2D correctly identified 97% of subjects with a mutation. The majority (438/597 (73%)) with other types of young-onset diabetes had an HbA1c above the upper limit of the age-appropriate *GCK* reference range. HbA1c ≥48 mmol/mol classified more people with *GCK* mutations as having diabetes than FPG ≥7 mmol/l (68% vs. 48%, p = 0.0009).

**Conclusions:**

Current HbA1c diagnostic criteria increase diabetes diagnosis in patients with a *GCK* mutation. We have derived age-related HbA1c reference ranges that can be used for discriminating hyperglycaemia likely to be caused by a *GCK* mutation and aid identification of probands and family members for genetic testing.

## Introduction

There is a contemporary drive to focus on haemoglobinA1c (HbA1c)[1], [2] rather than fasting plasma glucose (FPG) or oral glucose tolerance test (OGTT) in the diagnosis of diabetes. Maturity onset diabetes of the young (MODY) is a rare form of diabetes caused by a mutation in a single gene. Patients with MODY due to a heterozygous inactivating mutation in the *GCK* gene are frequently misdiagnosed with type 1 (T1D) or type 2 (T2D) diabetes yet they have lifelong mild fasting hyperglycaemia[3], [4], [5], [6], pharmacological treatment is rarely required [5], [7], [8] and the development of complications is atypical [8], [9], [10], [11], [12], [13]. These patients have traditionally been identified as suitable for genetic testing if they have a FPG in the range of 5.5–8.4 mmol/l or a small (<3 mmol/l) OGTT 2-hour increment [14].

The range of HbA1c has not been systematically studied in *GCK* families. The current recommendation that an HbA1c of ≥48 mmol/mol is diagnostic of diabetes could mean many more patients with *GCK* mutation related hyperglycaemia will be misdiagnosed with either T1D or T2D compared with using FPG or 2-hour OGTT values and be inappropriately treated.

We aimed to study HbA1c in families with a *GCK* mutation. The objectives were to 1) determine how effective HbA1c is as a discriminator between those with a mutation, those without a mutation (controls) and those with other young-onset diabetes; 2) to establish age-related HbA1c reference ranges in order to identify levels to help select those patients most likely to have hyperglycaemia due to a *GCK* mutation; 3) to investigate how effective upper and lower reference ranges are in distinguishing between GCK-MODY, controls and young-onset T1D and T2D and 4) to investigate whether changing from a glucose based diagnostic criteria to an HbA1c based diagnostic criteria will alter the proportion of patients with *GCK* mutations who are diagnosed with diabetes.

## Patients and Methods

With ethical approval (Devon and Torbay Research Ethics Committee, UK and the NHS Scotland Research Coordinating Centre, UK) we recruited and obtained written consent on individuals aged ≥18 years with a *GCK* mutation and familial controls. All GCK and control HbA1c measurements were performed at the Royal Devon and Exeter NHS Foundation Trust on a Tosoh (Tosoh Europe, Tessenderlo, Belgium) G8 anion exchange High Performance Liquid Chromatography (HPLC) analyser (Inter- and Intra assay CVs <3%).

HbA1c data were available for comparison from 278 patients with T1D and 319 patients with young onset T2D (diagnosed < = 35 y) from previous research[15].

To exclude coincidental T1D or T2D in patients with a *GCK* mutation outliers were removed using a robust outlier detection method[16] which is a standard technique used for determining normal biochemical ranges. For controls, current diagnostic ranges of an HbA1c and/or FPG were utilised[17].

Hba1c and FPG data were assessed for normality using histograms and the Shapiro-Wilk test. Comparisons between HbA1c and FPG values were assessed using the T-Test. Association of HbA1c and FPG with age was determined using regression analysis. Receiver Operating Characteristic (ROC) curves were used to analyse the effectiveness of HbA1c and FPG for discriminating between individuals with a mutation, controls and individuals with T1D or T2D.

Reference ranges for HbA1c and FPG were determined in both groups using mean +/−1.96 standard deviations (SD). Analysis was also split by age group (≤40 and >40 years) to account for age-related differences in glycaemia and to examine hyperglycaemia in young adults.

Density plots were used to show the distribution of Hba1c between the groups.

## Results

229 subjects were recruited from 55 families (47 different *GCK* mutations): 129 patients with *GCK* mutations and 100 familial controls. Six outliers with a *GCK* mutation were excluded based on an elevated HbA1c (66–79 mmol/mol (8.2–9.4%)). These patients also had additional features and characteristics supporting T2D in addition to a *GCK* mutation: obesity (median BMI 36.0, IQR 34.4–37.6), older age (median age 60.5 years, IQR 45.0–63.0), and high FPG (median 9.6 mmol/l, IQR 8.8–10.2). One control met HbA1c criteria of diabetes and was excluded due to likely T2D development: HbA1c 50 mmol/mol (6.7%), BMI 30.2, age 57 years.

### Baseline characteristics

The characteristics of the 123 individuals with a *GCK* mutation and 99 familial controls in this study were similar in age (median(IQR) 44 years(36–60) vs. 44 years(37–61), p = 0.5). Controls had a higher BMI (26.8 (23.9–30.2) vs. 25.8 (22.1–29.1), p = 0.01).

The characteristics of the 278 patients with T1D showed they were of a lower current age than those with a mutation (median(IQR) 37 years(11–51), p<0.001) but had comparable BMI (24.5 (22–28) p = 0.2). The 319 patients with young-onset T2D (diagnosed ≤35 years) were of similar age (42 years(36–50), p = 0.3) but had a higher BMI (33.0 (28–38), p<0.001).

### Glycaemic characteristics of patients with a GCK mutation and familial controls

**Figure 1:**
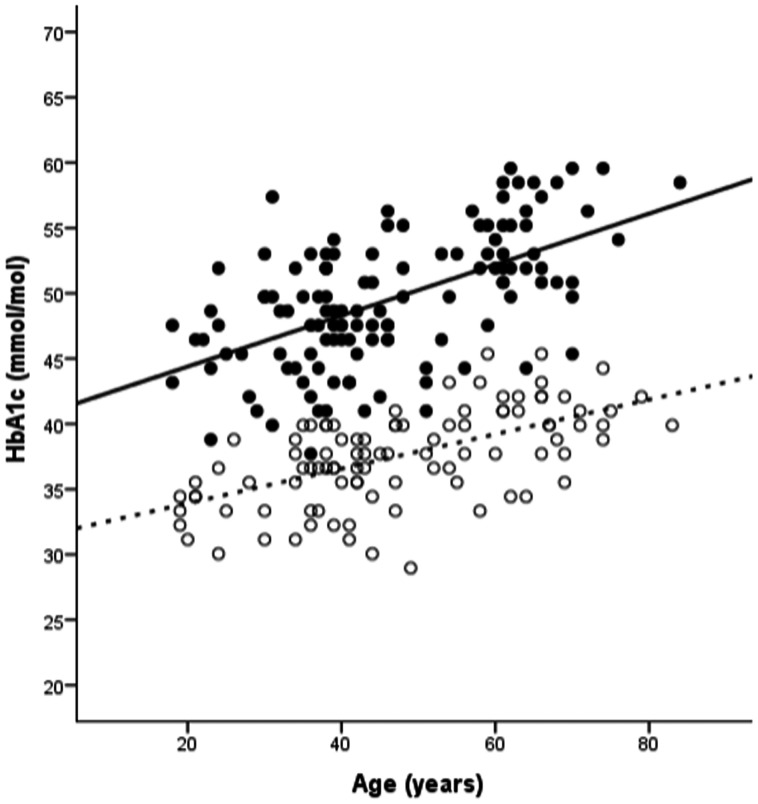
HbA1c increases with age in both GCK and controls. GCK indicated by black circles and black line, controls indicated by white circles and dashed line.

HbA1c values of *GCK* and control groups are shown in Figure 1. HbA1c was higher in patients with a *GCK* mutation compared with controls (mean(SD) 49 (5.0) mmol/mol (6.6%) vs. 38(3.6) (5.6%), p<0.001).

HbA1c significantly increased with age in both groups: GCK: 0.2 mmol/mol per year (95% CI 0.14–0.25, p<0.001); controls: 0.13 mmol/mol per year (95% CI 0.09–0.17, p<0.001), p = 0.06 for difference between *GCK* and control groups.

FPG was higher in patients with a *GCK* mutation than in controls (mean(SD) 6.9 mmol/l (0.75) vs. 5.0 mmol/l (0.52), p<0.001).

Those ≤40 years with a *GCK* mutation had an Hba1c reference range of 38–56 mmol/mol (5.6–7.3%) and those >40 had a reference range 41–60 mmol/mol (5.9–7.6%) (Table 1).

**Table 1:** HbA1c and FPG reference ranges in patients with *GCK* mutations and controls.

	Individuals *with* a GCK mutation		Individuals *without* a mutation (Controls)	
	≤40 years, n = 54	>40 years, n = 69	≤40 years, n = 36	>40 years, n = 63
**HbA1c%**, mean ±2 sd	47 mmol/mol (6.4%)	51 mmol/mol (6.8%)	36 mmol/mol (5.4%)	39 mmol/mol (5.7%)
**HbA1c mmol/mol**, mean ±2 sd	38–56 mmol/mol (5.6–7.3%)	41–60 mmol/mol (5.9–7.6%)	30–41 mmol/mol (4.9–5.9%)	32–46 mmol/mol (5.1–6.4%)
**HbA1c above diagnostic level** (≥48 mmol/mol)	56% (30/54)	78% (54/69)		
**FPG**, mean ±2 sd	6.8 mmol/l, 5.6–8.0 mmol/l	7.0 mmol/l, 5.4–8.7 mmol/l	4.9 mmol/l, 4.1–5.6 mmol/l	5.1 mmol/l, 4.0–6.3 mmol
**FPG above diagnostic level** (≥7.0 mmol/l)	41%, (22/54)	54%, (37/69)		
**HbA1c ** ***and*** ** FPG at diagnostic level**	43/123 (35%)			
**Neither HbA1c nor FPG at diagnostic level**	23/123 (19%)			

Data presented according to ≤40 and >40 year age groups and proportions diagnosed with diabetes using HbA1c ≥48 mmol/mol (6.5%) or FPG ≥7.1 mmol/l.

Abbreviations: FPG: fasting plasma glucose, HbA1c: haemoglobinA1c.

### The performance of HbA1c and FPG to identify GCK MODY from controls

**Figure 2:**
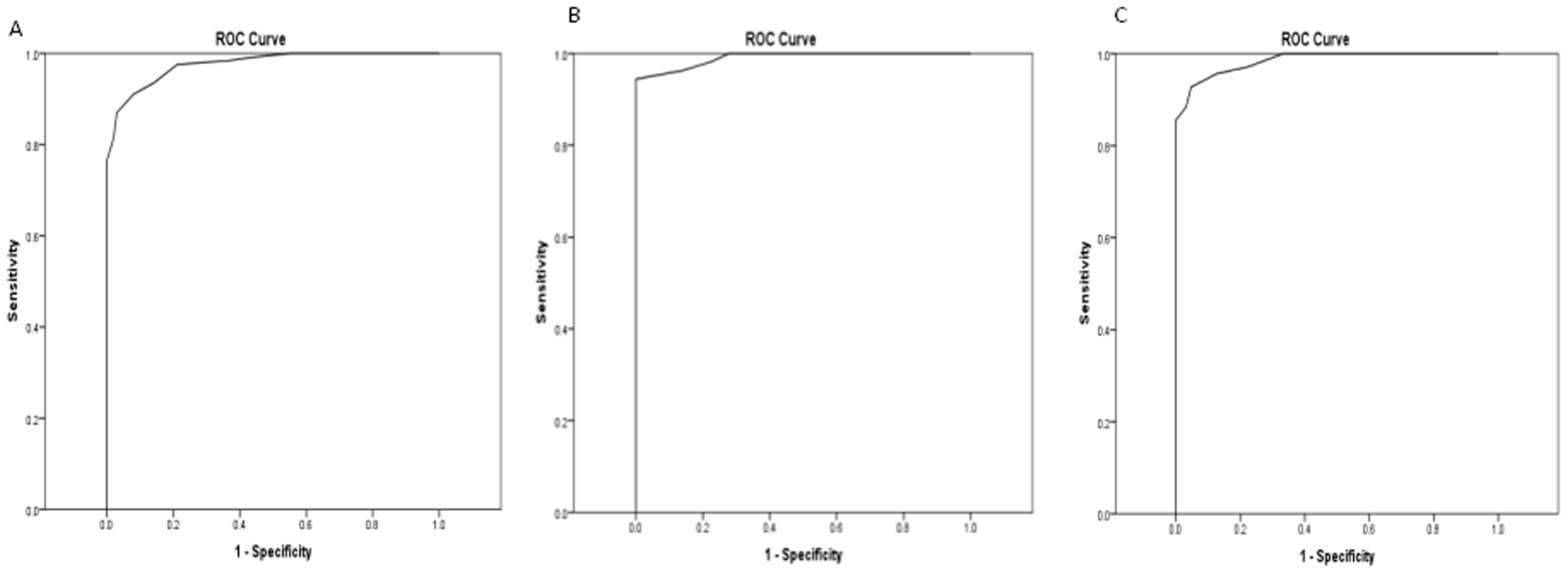
Receiver operator characteristic curves to identify mutation patients with a *GCK* mutation from controls using HbA1c. (A) all ages AUC 0.97 (B) ≤40 years of age AUC 0.99 (C) >40 years of age AUC 0.98.

**Figure 3:**
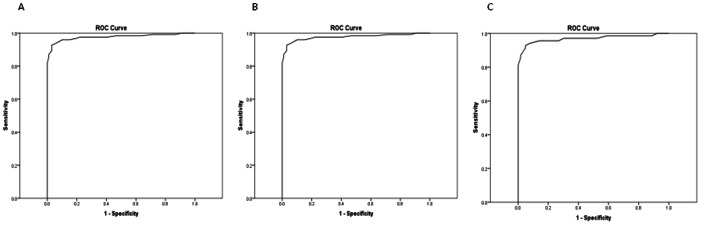
Receiver operator characteristic curves to identify mutation patients with a *GCK* mutation from controls using FPG. (A) all ages AUC 0.97 (B) ≤40 years of age AUC 0.98, (C) >40 years of age AUC 0.97.

HbA1c and FPG were effective for identifying individuals with a *GCK* mutation from controls: ROC Area Under the Curve (AUC) 0.97 for both. This was similar when splitting data into 2 age groups: ≤40 years and >40years (figures 2 and 3).

All patients (123/123) with a *GCK* mutation were above the lower limit of the HbA1c age-appropriate reference ranges (≥38 mmol/mol (5.6%)) in those aged ≤40 years and in those >40 (≥41 mmol/mol (5.9%)). 69% (31/99) of controls were below these lower limits indicating a diagnosis of a *GCK* mutation could be correctly ruled out in the majority of cases.

97% (119/123) of patients with a *GCK* mutation were above the lower limit of the FPG age-appropriate reference ranges (≥5.6 mmol/l) in those aged ≤40 years and in those aged >40 years (≥5.4 mmol/l). 81% (80/99) of controls were below these lower limits indicating a diagnosis of a *GCK* mutation could be correctly ruled out in the majority of cases.

### The performance of HbA1c to identify GCK MODY from young-onset type 1 and type 2 diabetes

**Figure 4:**
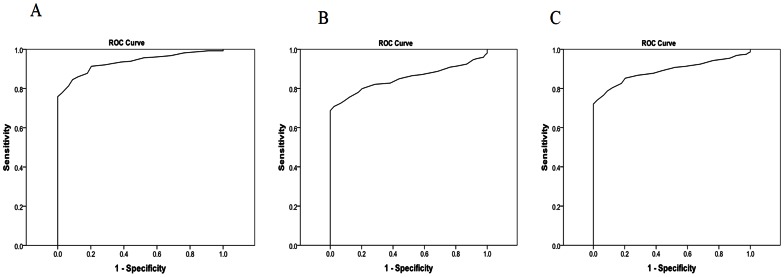
Receiver operator characteristic curves to identify mutation patients with a *GCK* mutation from those with type 1 or type 2 diabetes using HbA1c. (A) mutation carrying patients from those with type 1 diabetes AUC 0.94 (B) mutation carrying patients from those with type 2 diabetes AUC 0.86 and (C) mutation carrying patients from those with type 1 or type 2 diabetes (types combined) AUC 0.89.

HbA1c was also effective for identifying individual with a *GCK* mutation from those with T1D or T2D (AUC 0.94 and 0.86 respectively) and between those with a *GCK* mutation and T1D and T2D combined together (AUC 0.89, figure 4).

**Figure 5:**
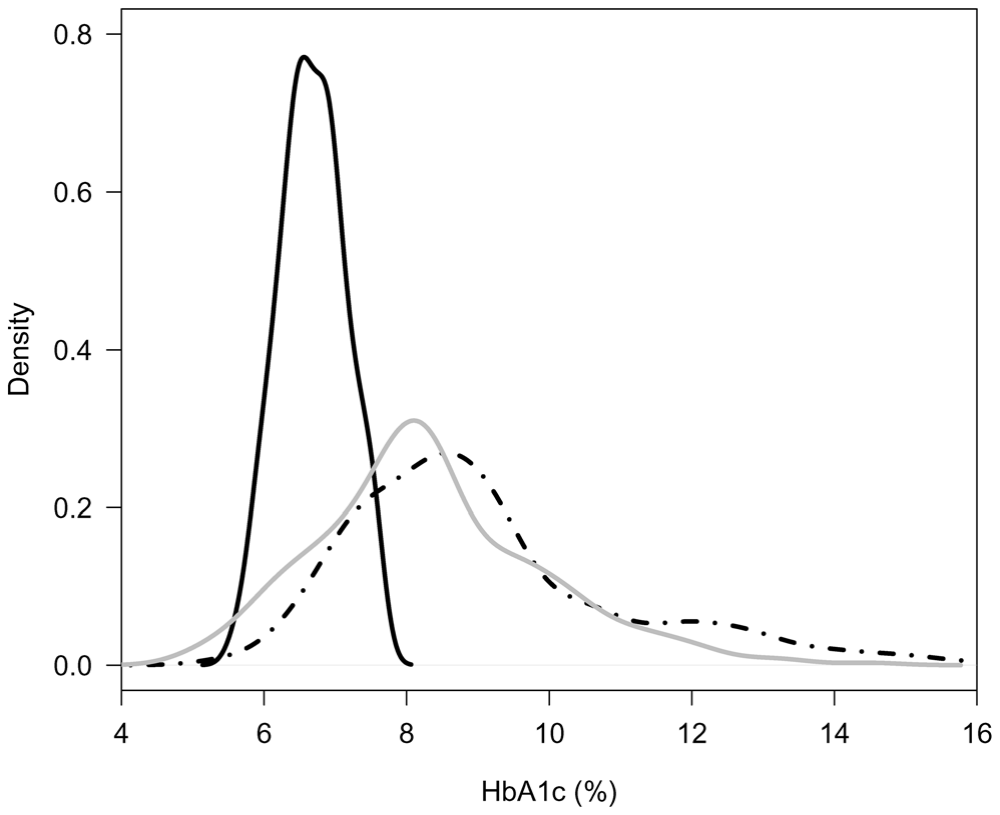
Density plots to show the distribution of HbA1c results for GCK, type 1 and type 2 diabetes. GCK  =  solid black line, type 1 =  dashed line and type 2 =  grey solid line. To convert values for HbA1c in% into mmol/mol, subtract 2.15 and multiply by 10.929.

The distributions of HbA1c results for patients with a *GCK* mutation and patients with T1D and T2D are shown in Figure 5.

Using the upper limit of the age-appropriate reference ranges (56 mmol/mol (7.3%)) for those aged ≤40 years (60 mmol/mol (7.6%)) and for those aged >40years to discriminate those with a *GCK* mutation from those with T1D/T2D correctly identified 97% of subjects with a mutation. The majority (438/597 (73%)) of those with other types of young-onset diabetes had an HbA1c above the upper limit of the age-appropriate GCK reference range indicating a *GCK* mutation could appropriately be ruled out in these cases.

### Prevalence of diabetes in GCK MODY using current diabetes diagnostic ranges

Current diagnostic criteria of an HbA1c ≥48 mmol/mol (6.5%) classifies more patients with *GCK* mutations as having diabetes than a FPG ≥7 mmol/l (68% (84/123) v 48% (59/123) respectively, p = 0.001).

41/84 (49%) of those classified as having diabetes based on HbA1c would not have been classified with diabetes based on FPG. Conversely, 16/59 (27%) diagnosed using FPG would not be diagnosed with diabetes based on their HbA1c (Table 1).

## Discussion

This study is the first to identify reference ranges of HbA1c for patients with a *GCK* mutation. The discriminatory accuracy of HbA1c between these groups was high (ROC AUC∼0.9) and the reference ranges provided good sensitivity and specificity for aiding the identification of individuals likely to have a mutation compared with both controls and those with T1D or T2D.

### HbA1c to identify GCK MODY

Traditionally, FPG or OGTT have been useful in identifying patients with a *GCK* mutation within families or when choosing patients for genetic testing [14], but given that diagnostic criteria for diabetes are changing to place emphasis on HbA1c it was important to investigate this as an alternative measure. Just two studies have previously reported HbA1c. These were undertaken prior to the standardisation of biochemical analysis for HbA1c and performed on smaller cohorts of 18 and 56 individuals with a mutation [8], [11]. We provide robust and widely generalisable HbA1c ranges in these patients. For our patients with a *GCK* mutation and our controls, we used the same laboratory for biochemical analysis and used a method that is certified by the National Glycohemoglobin Standardization Program [17].

Previous work has shown it is likely that patients have a *GCK* mutation if they are found to have incidental hyperglycaemia in the paediatric age [3] whereas in cohorts diagnosed later, *GCK* mutations are much rarer [18], [19]. The lower limits of HbA1c observed in our cohort with a *GCK* mutation (38 mmol/mol (5.6%) ≤40 years, 41 mmol/mol (5.9%) >40 years) are useful to identify whether individuals within *GCK* families should have genetic testing. The upper limits (56 mmol/mol (7.3%) ≤40 years, 60 mmol/mol (7.6%) >40 years) are useful for distinguishing which patients are very unlikely to have a *GCK* mutation and so their diabetes can be attributed to other subtypes. The higher limits are also useful for considering whether older patients with *GCK* mutations have developed concurrent T2D.

### Use of age-related HbA1c reference ranges for discriminating patients with a GCK mutation from controls

HbA1c showed excellent discrimination between patients with a *GCK* mutation and familial controls (ROC AUC = 0.97) and the age-related references ranges were useful in correctly ruling out a *GCK* mutation in 69% of those who were unaffected. This indicates that HbA1c is a very useful clinical measure when determining which other members of an affected family should be tested. Using age-related reference ranges of HbA1c alongside best practice guidelines for the molecular genetic diagnosis of MODY[20] will help identify appropriate subjects for genetic testing within known GCK families. Our data add to these guidelines but we do not suggest that the values are used as absolute cut-offs or in isolation. Our reference ranges provide a practical guide to be used alongside a patient's clinical features.

### Use of age-related HbA1c reference ranges for discriminating GCK from other forms of young-onset diabetes

HbA1c showed excellent discrimination between patients with a *GCK* mutation and patients with other young-onset diabetes with an ROC AUC = 0.89. This is comparable with other biochemical biomarkers used in the discrimination of HNF1A-MODY from other diabetes (e.g. hs-CRP (high sensitivity C-reactive protein)[21], [22], [23] and 1,5Ag (serum 1,5 anhydroglucitol)[24]) which both have ROC AUC ∼0.8. In addition, the benefit of using HbA1c is clear in that it is a clinically straightforward option as it is measured routinely.

The majority of patients (73%) with either T1D or young-onset T2D had HbA1cs above the *GCK* reference ranges allowing them to not be considered for *GCK* mutation testing. Some patients with T1D or T2D have excellent control but this is not surprising given that intensity of therapy and extent of progressive beta cell dysfunction determines HbA1c in these types of diabetes. In discriminating *GCK* from these two other common types of diabetes with excellent control, other features indicative of GCK-MODY should be sought including a young age of diagnosis, the individual having an affected parent (but this may require testing), no pharmaceutical treatment, no deterioration in glycaemic control with reduction or omission of glucose lowering therapy and lack of microvascular complications in affected family members [8], [10], [11], [15], [25].

### Impact of using FPG or HbA1c for diabetes diagnosis in GCK MODY

In known *GCK* families, either FPG or HbA1c may be used to distinguish between individuals likely or unlikely to have a mutation. However, the care and screening provided for patients with a mutation should be based on their clinical characteristics and should not vary if the HbA1c or fasting glucose is just above or just below the criteria for diabetes either by FPG or HbA1c.

Previous work has shown that 38% of patients with a *GCK* mutation would be classified as having diabetes using FPG alone [14] (>7.0 mmol/l[26]) and that only 24% of these would also have a 2-hour OGTT value in the range where diabetes was diagnosed [14]. Population screening for diabetes now frequently uses HbA1c rather than FPG. Our results suggest that more individuals with a *GCK* mutation will be diagnosed with diabetes using an HbA1c ≥48 mmol/mol (6.5%) rather than using FPG of ≥7.1 mmol/l (68% vs. 48%). Using both HbA1c *and* FPG would mean only 19% of subjects with the *GCK* mutations would be diagnosed with diabetes.

If the correct diagnosis of a *GCK* mutation is not made, patients may receive unnecessary treatment for hyperglycaemia, more aggressive treatment of risk factors such as blood pressure and cholesterol and an increase in screening for complications. Since studies to date have not established that patients with a *GCK* mutation have an increased risk of macrovascular disease (8, 10, 11), such treatment is unlikely to be beneficial. Serious microvascular complications have not been reported in children and young adults with a mutation and they are very rare even after a lifetime of untreated *GCK* related hyperglycaemia (8–11, [12], [13].

It is important that those with stable, mild hyperglycaemia (either by FPG or HBA1c) be considered for *GCK* mutation testing to prevent inappropriate management as T1D or

T2D. This should also avoid the adverse impact of making a diagnosis of diabetes on insurance and employment opportunities.

### Limitations

No patient had diseases known to affect the measurement of HbA1c [2] but only haemoglobinopathies were screened for in our study. Iron deficiency anaemia was not systematically excluded but this probably reflects routine practice.

2-hour OGTT data were only available for 49/222 (22%) subjects therefore outliers were removed using a robust outlier detection method (patients with a *GCK* mutation) and current diagnostic HbA1c and FPG values (controls).

## Conclusion

With the current trend for measuring HbA1c rather than FPG, we demonstrate how HbA1c measured in families with a heterozygous inactivating mutation *GCK* mutation can be used to identify those suitable for diagnostic molecular genetic testing.

Alongside clinical features, the age-related reference ranges provided are effective for identifying the likely mutation status of individuals within a known *GCK* family prior to genetic testing and for excluding the need for *GCK* mutation testing in other individuals referred for molecular genetic testing.

The use of HbA1c has become the diagnostic measure of choice for diabetes in many countries but, its use will increase the proportion of patients with a *GCK* mutation related hyperglycaemia being misdiagnosed with diabetes.
